# Uptake Study in Lysosome-Enriched Fraction: Critical Involvement of Lysosomal Trapping in Quinacrine Uptake but Not Fluorescence-Labeled Verapamil Transport at Blood-Retinal Barrier

**DOI:** 10.3390/pharmaceutics12080747

**Published:** 2020-08-08

**Authors:** Yoshiyuki Kubo, Miki Yamada, Saki Konakawa, Shin-ichi Akanuma, Ken-ichi Hosoya

**Affiliations:** Department of Pharmaceutics, Graduate School of Medicine and Pharmaceutical Sciences, University of Toyama, 2630 Sugitani, Toyama 930-0194, Japan; s1560205@ems.u-toyama.ac.jp (M.Y.); s1560221@ems.u-toyama.ac.jp (S.K.); akanumas@pha.u-toyama.ac.jp (S.-i.A.)

**Keywords:** blood-retinal barrier, cationic drug, transport, lysosomal trapping

## Abstract

Lysosomal trapping at the blood–retinal barrier (BRB) was investigated through quinacrine and fluorescence-labeled verapamil (EFV) uptake. Quinacrine uptake by conditionally immortalized rat retinal capillary endothelial (TR-iBRB2) cells suggested saturable and non-saturable transport processes in the inner BRB. The reduction of quinacrine uptake by bafilomycin A1 suggested quinacrine distribution to the acidic intracellular compartments of the inner BRB, and this notion was also supported in confocal microscopy. In the study using the lysosome-enriched fraction of TR-iBRB2 cells, quinacrine uptake was inhibited by bafilomycin A1, suggesting the lysosomal trapping of quinacrine in the inner BRB. Pyrilamine, clonidine, and nicotine had no effect on quinacrine uptake, suggesting the minor role of lysosomal trapping in their transport across the inner BRB. Bafilomycin A1 had no effect on EFV uptake, and lysosomal trapping driven by the acidic interior pH was suggested as a minor mechanism for EFV transport in the inner BRB. The minor contribution of lysosomal trapping was supported by the difference in inhibitory profiles between EFV and quinacrine uptakes. Similar findings were observed in the outer BRB study with the fraction of conditionally immortalized rat retinal pigment epithelial (RPE-J) cells. These results suggest the usefulness of lysosome-enriched fractions in studying lysosomal trapping at the BRB.

## 1. Introduction

The blood–retinal barrier (BRB) has two barrier structures, the inner and outer BRB, separating the neural retina and circulating blood [1,2,3]. The study of transport mechanisms at the BRB is assumed to be essential for improving drug therapy of retinal diseases because the efficient and safe delivery of drugs to the retina is a major challenge [1,2,3]. The retinal capillary endothelial and pigment epithelial cells are main constituents of the inner and outer BRB, respectively [3]. These cells are largely responsible for the transcellular blood-to-retina transport of nutrients and drugs, and the paracellular transport is limited by tight junctions [3]. Previous progress in the study of the BRB achieved using in vivo and in vitro methods has demonstrated the critical roles of blood-to-retina transport across the BRB in the retinal homeostasis [2,3]. Furthermore, these studies have clearly shown that nutrient transport at the BRB involves various membrane transporters, such as glucose transporter (GLUT1/SLC2A1), taurine transporter (TAUT/SLC6A6), creatine transporter (CRT/SLC6A8), cationic amino acid transporter (CAT1/SLC7A1), equilibrative nucleoside transporter (ENT2/SLC29A2), and riboflavin transporter (RFVTs/SLC52A) [3,4,5,6,7,8,9,10,11].

In addition, recent advances in the study of the BRB have revealed the involvement of novel transport systems in the blood-to-retina transport of cationic drugs, such as pyrilamine, nicotine, propranolol, clonidine and verapamil at the inner and outer BRB [12,13,14,15,16,17]. These transport systems are considered to be promising in the efficient and safe delivery of drugs to the retina, because they recognize cationic drugs, such as desipramine, imipramine, memantine, and clonidine, whose neuroprotective effects on cerebral ischemia and optic nerve injury have been reported [18,19,20]. Therefore, the combination of neuroprotective cations and transport systems at the BRB is expected to be beneficial in the effective therapy of neurological dysfunction of the retina, such as macular degeneration and diabetic retinopathy with severe symptoms, including blindness [3,12].

Furthermore, functional studies have revealed the temperature-dependent and saturable characteristics of the carrier-mediated cationic drug transport systems at the inner BRB, suggesting that they involve unknown membrane transporters expressed at the BRB [12,13,14,15,16,17]. In addition, cumulative evidence demonstrates that the substrate candidates of the systems include several cationic amphiphilic drugs that possibly undergo lysosomal trapping and are sequestrated in lysosomes, which are acidic organelles [21,22,23]. Uptake studies using a typical lysosomotropic agent, LysoTracker^®^ Red DND-99 (LysoTracker Red; LTR, p*Ka* = 7.5, Log *P* = 2.1) [23,24,25], suggested that lysosomal trapping takes place in the retinal capillary endothelial cells (inner BRB) and that the uptake of LTR involves saturable and non-saturable transport processes in the inner BRB [26]. Inhibition studies suggested that lysosomal trapping has a minor effect on the transport of clonidine, nicotine, pyrilamine and verapamil at the inner BRB [26]. Furthermore, this suggestion was also supported by in vivo and in vitro studies with a fluorescence-labeled verapamil, EverFluor FL verapamil (EFV), which clearly demonstrated the permeation of verapamil from circulating blood to the retina [27].

However, further studies are still essential to determine the influence of lysosomal trapping in the BRB because it has been reported to significantly affect cationic drug distribution in the lung and liver [28,29,30]. In the present study, lysosomal trapping at the BRB was investigated using quinacrine as a test compound because it is suggested to be a lysosomotropic agent of antimalaria with intrinsic fluorescence by its physicochemical properties (p*Ka* = 10.3, Log *P* = 5.2). The accumulation of quinacrine in acidic intracellular compartments was reported to be inhibited by bafilomycin A1, a specific inhibitor of vacuolar (V)-ATPase [31,32,33], supporting the notion that quinacrine is a useful test compound for the study of lysosomal trapping. In the uptake study, we used the lysosome-enriched fraction prepared from the in vitro model cell line of the inner and outer BRB, which was also tried for EFV to investigate the influence of lysosomal trapping on the blood-to-retina transport of cationic drugs at the BRB.

## 2. Materials and Methods

### 2.1. Materials

LTR, EFV and quinacrine were purchased from Thermo Fisher Scientific (Waltham, MA, USA), Setareh Biotech (Eugene, OR, USA) and Merk (St. Louis, MO, USA), respectively. Optiprep^TM^, an iodixanol solution for the purification of biological particles, was obtained from Abbott Diagnostics Technologies AS (Oslo, Norway), and protease inhibitor cocktail was purchased from Merk. Conditionally immortalized rat retinal capillary endothelial (TR-iBRB2) cells and rat retinal pigment epithelial (RPE-J) cells were established by Hosoya et al and Nabi et al, respectively [34,35], and these were used as in vitro model cell lines of inner and outer BRB, respectively. The fetal bovine serum (FBS) used for these cultures was purchased from SAFC Bioscience (Lenexa, KS, USA). Chemicals of reagent-grade used in this study were commercially obtained, and the supplemental information (Supplementary Materials) shows the buffer composition. The bafilomycin A1 used to study the effect of acidic interior pH was obtained from LC Laboratories (Woburn, MA, USA), while ammonium chloride (NH_4_Cl) and carbonyl cyanide-p-trifluoromethoxyphenylhydrazone (FCCP) were obtained from Merk.

### 2.2. Methods

#### 2.2.1. Cellular Uptake Analysis

Cellular uptake analysis was designed as described in our previous reports [13,14,15,16]. BioCoat^TM^ Collagen I Cellware 24-well culture dishes (Corning, Corning, NY, USA) were used for TR-iBRB2 cells. Before the assay, the cells were rinsed with extracellular fluid (ECF)-buffer warmed to 37 °C. The uptake assay was initiated by adding ECF-buffer containing 5 μM test compound, and was terminated by washing with ice-cold ECF-buffer three times. The fluorescent intensity of test compounds taken up by the cell was determined using SpectraMax i3 microplate detection system (Molecular Devices, San Jose, CA, USA) after cell disruption using an ultrasonic homogenizer. The cellular uptake was expressed as the cell-to-medium ratio (cell/medium) calculated using the following equation:

Cell/medium ratio = (fluorescence intensity in the cells)/(fluorescence intensity in the medium)(1)

The nonlinear least-squares regression analysis program (MULTI) was used for data analysis [36], and kinetic parameters, such as the Michaelis constant (K_m_), maximal uptake rate (V_max_), and non-saturable uptake rate (K_d_), for cell uptake were estimated by fitting data obtained in the cellular uptake analysis to the following equation, where the test compound concentration and uptake rate are S and V, respectively.

V = (V_max_ × S)/(K_m_ + S) + K_d_ × S (2)

#### 2.2.2. Confocal Microscopy of Fluorescent Compounds

The fluorescence distribution in TR-iBRB2 cells was analyzed as described in our previous report, and BioCoat™ Collagen I eight-well culture slides were used for culturing TR-iBRB2 cells (4500 cells/slide) for 48 hr at 33 °C [27,37]. Prior to confocal microscopy, the cells were incubated in ECF-buffer containing the test compound for 30 min. The incubation was terminated by washing the cells with ice-cold ECF-buffer, and cells were treated with phosphate-buffered saline (PBS) containing 4% paraformaldehyde (PFA) for 20 min in the dark. The slides were treated with VECTASHIELD mounting medium (Vector Laboratories, Burlingame, CA, USA), followed by confocal microscopy using a TCS-SP5 confocal microscope (Leica Microsystems, Wetzlar, Germany).

#### 2.2.3. Preparation of Lysosome-Enriched Fraction

BioCoat^TM^ Collagen I Cellware 10 mm dishes (Corning) were used for culturing TR-iBRB2 and RPE-J cells, and the lysosome-enriched fractions were prepared as described in our previous reports [7,8,38]. Cells were rinsed with PBS and collected in suspension buffer (25 mM sucrose, 10 mM HEPES, 1 mM ethylenediaminetetraacetic acid (EDTA), 0.1% protease inhibitor cocktail, pH 7.4) using a cell lifter (Corning), followed by centrifugation for 5 min at 200× g and 4 °C. The cells were suspended in suspension buffer, homogenized using a Teflon glass homogenizer (2500 rpm, 10 strokes), and then centrifuged for 10 min at 800× g at 4 °C. The resultant post nuclear supernatant was collected and layered on top of a discontinuous gradient composed of 12%, 15%, and 18% iodixanol (Optiprep^TM^) in suspension buffer, followed by centrifugation for 3 h at 178,000× g at 4 °C in a near-vertical rotor, NVT65, and Optima L70 (Beckman Coulter, Brea, CA). The gradient solutions were collected into eleven fractions using 18G syringe needles, and their densities were determined using a digital refractometer (Atago, Tokyo, Japan). The fractions were also assayed for acid phosphatase activity for the isolation of the lysosome-enriched fraction and *p*-nitrophenylphosphate was used as a substrate to measure *p*-nitrophenol, a reaction product, using a GeneQuant 1300 spectrophotometer (GE Healthcare, Chicago, IL, USA).

#### 2.2.4. Uptake Assay of Cationic Drugs in Lysosome-Enriched Fraction

The lysosome-enriched fraction was rinsed in intracellular fluid (ICF)-buffer (140 mM K-gluconate, 4 mM NaCl, 2 mM K_2_HPO_4_, 2 mM MgCl_2_, 0.39 mM CaCl_2_, 1 mM ethylene glycol tetraacetic acid (EGTA), and 10 mM HEPES, pH 7.2) by centrifuging twice for 30 min at 20,000× *g* at 4 °C to remove the suspension buffer [38,39]. The assay was initiated by suspending the lysosome-enriched fraction in 100 μM ICF-buffer containing the test compounds at 37 °C and was terminated by adding 1000 μL ice-cold ICF-buffer. The assay mixture was centrifuged for 30 min at 20,000× *g* and 4 °C, and the resultant pellet was solubilized by adding 100 μL 1% Triton X-100 for 8 h, followed by fluorescence determination at excitation and emission wavelengths (λ_ex_ and λ_em_, respectively) of 420 nm and 500 nm for quinacrine and 480 nm and 510 nm for EFV, respectively using SpectraMax i3 microplate detection system. The uptake in the lysosome-enriched fraction was expressed as fraction-to-medium ratio (fraction/medium) calculated using the following equation:

Fraction/medium ratio = (fluorescence intensity in the lysosome-enriched fraction) /(fluorescence intensity in the medium)(3)

In addition, the inhibition in uptake by the lysosome-enriched fraction was expressed as relative uptake (percentage of control, %) calculated with the following equation.

Relative uptake (%) = (fraction/medium ratio in the presence of inhibitor) /(fraction/medium ratio in the absence of inhibitor)(4)

#### 2.2.5. Statistical Analysis

In the determination of significant difference for two groups and for several groups was carried out using an unpaired two-tailed Student’s t-test was taken for two groups and a one-way analysis of variance (ANOVA) followed by Dunnett’s test, respectively. In the least-square regression analysis, the kinetic parameter was expressed as mean values ± standard deviation (S.D.). Unless otherwise indicated, data represent mean values ± standard error of the mean (S.E.M.).

## 3. Results

### 3.1. Uptake of Quinacrine by TR-iBRB2 Cells

**Figure 1:**
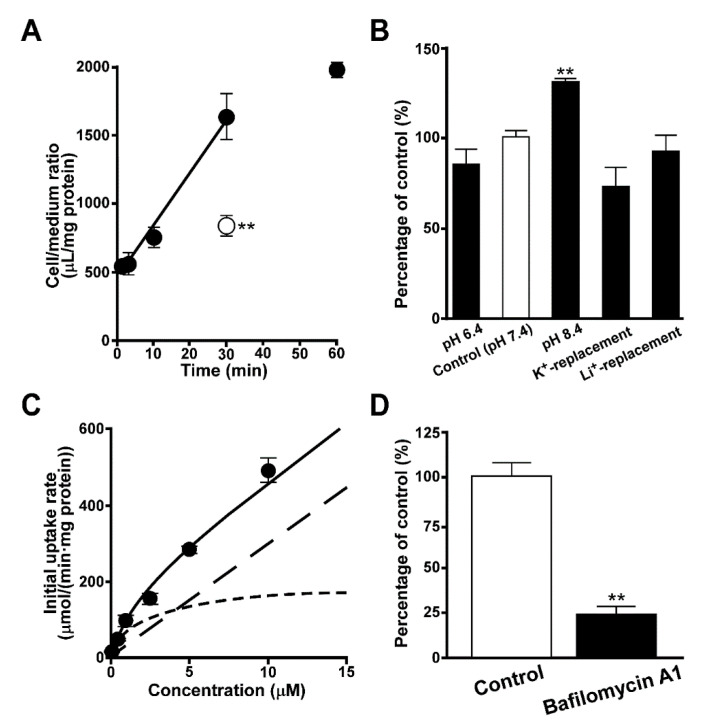
Quinacrine uptake by TR-iBRB2 cells. (**A**) Time-dependent uptake of quinacrine (5 μM) by TR-iBRB2 cells was investigated at 37 °C (closed circles) and 4 °C (open circles). (**B**) The effects of Na^+^, extracellular pH and membrane potential were examined at 37 °C for 30 min. (**C**) Concentration-dependent uptake was examined at 37 °C for 30 min, over the concentration range 1–10 μM. Dotted and dashed lines represent a saturable and non-saturable transport process, respectively. (**D**) The effects of bafilomycin A1 was examined. Before quinacrine uptake at 37 °C for 30 min, cells were treated with 100 nM bafilomycin A1 at 37 °C for 30 min. Each column and point represent the mean ± S.E.M. (n = 3–4). Significant difference for two groups and for several groups was taken using an unpaired two-tailed Student’s t-test was taken for two groups and a one-way analysis of variance (ANOVA) followed by Dunnett’s test, respectively. ** *p* < 0.01, significantly different from the control (37 °C, pH 7.4).

The uptake of quinacrine was examined using TR-iBRB2 cells as the in vitro model cell line of the inner BRB. The time-course study showed a time-dependent linear increase of quinacrine at 37 °C after at least 30 min (Figure 1A), and the initial uptake rate was calculated to be 445 ± 51 µL/(min·mg protein). The uptake of quinacrine at 30 min was calculated to be 1640 ± 170 µL/(min·mg protein), which was 540 times greater than the cellular volume (≈3 µL/{mg protein}). In TR-iBRB2 cells, quinacrine uptake was significantly reduced by 45% at 4 °C (Figure 1A), and significantly increased by 31% at pH 8.4, while the uptake exhibited no significant change in K^+^- or Li^+^-replacement buffer (Figure 1B).

In the study of concentration-dependence, quinacrine uptake by TR-iBRB2 cells exhibited a saturable process with K_m_ and V_max_ values of 1.87 ± 0.61 μM and 189 ± 50 μmol/(min·mg protein), respectively, and a non-saturable process with a K_d_ value of 29.7 ± 3.0 μL/(min·mg protein) (Figure 1C). The contribution of saturable and non-saturable processes was estimated to be 52% and 48%, respectively at a concentration of 5 μM. In addition, pretreatment of TR-iBRB2 cells with bafilomycin A1 significantly reduced quinacrine uptake by 76% (Figure 1D).

### 3.2. Effect of Intracellular pH on Quinacrine Uptake by TR-iBRB2 Cells

**Figure 2:**
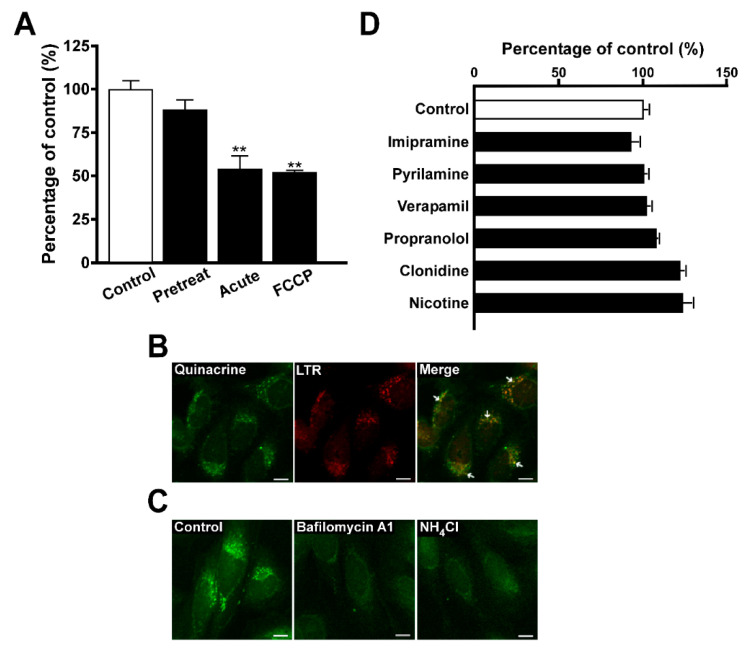
Effect of intracellular pH modulators on quinacrine uptake by TR-iBRB2 cells. (**A**) The quinacrine uptake was examined in the presence or absence of 30 mM NH_4_Cl at 37 °C for 30 min. In a pretreat condition, cells were treated with 30 mM NH_4_Cl, and the uptake of quinacrine (5 μM) was performed in the absence of NH_4_Cl. In an acute condition, cells were treated with ECF-buffer, and quinacrine uptake was performed in the presence of 30 mM NH_4_Cl. The uptake was also examined in the presence of 50 μM FCCP at 37 °C for 30 min. (**B**) Confocal microscopy was carried out to investigate the intracellular distribution of quinacrine and LTR. The uptake of quinacrine (5 μM, green) and LTR (300 nM, red) was carried out at 37 °C for 30 min. (**C**) Confocal microscopy was carried out to investigate the intracellular distribution of quinacrine in the presence of intracellular pH modulator, such as 100 nM bafilomycin A1 and 30 mM NH_4_Cl. The uptake of quinacrine (5 μM, green) was carried out at 37 °C for 30 min. (**D**) Inhibitory effect of cationic drugs on quinacrine uptake was examined in the presence of bafilomycin A1. After the treatment of TR-iBRB2 cells with 100 nM bafilomycin A1 at 37 °C for 30 min, quinacrine uptake was examined in the presence of 100 μM cationic drug at 37 °C for 30 min. Each column represents the mean ± S.E.M. (*n* = 3–4). ** *p* < 0.01, significantly different from the control. FCCP, Carbonyl cyanide-*p*-trifluoromethoxyphenylhydrazone. Scale bar, 10 μm.

The uptake of quinacrine at various intracellular pH conditions was examined in TR-iBRB2 cells, and acute treatment with NH_4_Cl reduced it by 47% while pretreatment with NH_4_Cl had no significant effect on the uptake (Figure 2A). In addition, quinacrine uptake by TR-iBRB2 cells was reduced by 48% in the presence of FCCP, a H^+^ ionophore (Figure 2A). Confocal microscopy showed the punctate fluorescence signal of quinacrine (green) taken up by TR-iBRB2 cells, and its intracellular distribution was similar to that of LTR (red), a typical compound that undergoes lysosomal trapping (Figure 2B). In addition, the punctate fluorescence signal of quinacrine was attenuated by treatment with bafilomycin A1 or NH_4_Cl (Figure 2C).

### 3.3. Inhibition of Quinacrine Uptake by TR-iBRB2 Cells

The inhibitory effect of compounds on the uptake of quinacrine by TR-iBRB2 cells was examined. The uptake was significantly decreased by various cationic drugs such as desipramine, imipramine, propranolol, verapamil, pyrilamine and nicotine, but was not changed by *p*-aminohippuric acid (PAH), choline, L-carnitine and cimetidine (Table 1). When TR-iBRB2 cells were treated with 100 nM bafilomycin A1, no significant effect was observed in the uptake of quinacrine in the presence of imipramine, amiodarone, pyrilamine, verapamil, propranolol, clonidine, and nicotine (Figure 2D).

### 3.4. Uptake of Quinacrine in Lysosome-Enriched Fraction

**Figure 3:**
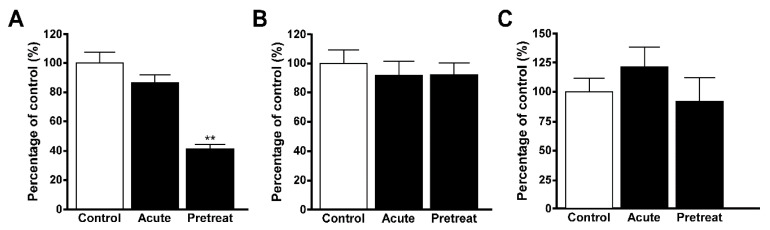
Effect of lysosomal pH on quinacrine and EFV uptake in lysosome-enriched fractions. (**A**) The uptake of quinacrine (5 μM) in the fraction of TR-iBRB2 cells was examined in the presence or absence of 100 nM bafilomycin A1 at 37 °C for 30 min. (**B**) The uptake of EFV (1 μM) in the fraction of TR-iBRB2 cells was examined in the presence or absence of 100 nM bafilomycin A1 at 37 °C for 30 min. (**C**) The uptake of EFV (1 μM) in the fraction of RPE-J cells was examined in the presence or absence of 100 nM bafilomycin A1 at 37 °C for 30 min. Bafilomycin A1 is an inhibitor of V-ATPase that attenuate the lysosomal acidic interior pH, and the uptake was examined in the presence or absence of 100 nM bafilomycin A1 at 37 °C for 30 min. In a pretreat condition, fractions were treated with 100 nM bafilomycin A1, and the uptake was performed in the absence of bafilomycin A1. In an acute condition, fractions were treated with ICF-buffer, and the uptake was performed in the presence of 100 nM bafilomycin A1. Each column represents the mean ± S.E.M (*n* = 4). ** *p* < 0.01, significantly different from the control.

The uptake of quinacrine in the lysosome-enriched fraction of TR-iBRB2 cells was significantly reduced by 53%, 50%, 34%, and 58% in the presence of desipramine, imipramine, propranolol and verapamil, respectively whereas pyrilamine, clonidine and nicotine had no effect (Table 1). The uptake of quinacrine in the lysosome-enriched fractions of TR-iBRB2 cells was examined following pretreatment with 100 nM bafilomycin A1, which significantly reduced the uptake (Figure 3A). The uptake of quinacrine in lysosome-enriched fractions of RPE-J cells was also examined, and it was significantly reduced by 36%, 38%, 23%, 43%, and 34% in the presence of desipramine, imipramine, propranolol, verapamil, and pyrilamine, respectively, whereas clonidine and nicotine had no effect (Table 1).

### 3.5. Uptake of EFV in Lysosomal-Enriched Fraction

The uptake of EFV was examined in the lysosome-enriched fractions of TR-iBRB2 cells, and desipramine significantly reduced it by 38%. Imipramine and verapamil also reduced the uptake by 32% and 28%, respectively, indicating their tendency to inhibit the uptake, whereas no effect was shown by propranolol, pyrilamine, clonidine and nicotine (Table 2). The uptake of EFV by lysosome-enriched fractions of RPE-J cells was significantly reduced by 35% and 49% in the presence of desipramine and verapamil, respectively, whereas no effect was shown by imipramine, propranolol, pyrilamine, clonidine and nicotine (Table 2). EFV uptake was also examined in the presence of bafilomycin A1, and no significant alteration was observed in the lysosome-enriched fraction of TR-iBRB2 and RPE-J cells in the presence of 100 nM bafilomycin A1 (Figure 3B,C).

## 4. Discussion

P-glycoprotein (P-gp/Mdr1/Abcb1) is known to be a representative efflux transporter at the blood-brain barrier, and involved in restricting the distribution of lipophilic and cationic drugs to the brain [40]. P-gp was also reported to be localized at the luminal membrane of the retinal capillary endothelial cells [41], suggesting its possible contribution to the restriction of drug distribution to the retina. However, the study of relationship between the BRB permeability and the lipophilicity of compounds showed a greater in vivo retinal uptake of verapamil, a substrate of P-glycoprotein [42], revealing the influx transport of verapamil at the BRB and the different barrier function between the BRB and the blood-brain barrier (BBB). Furthermore, the carrier-mediated blood-to-retina transport of cationic drugs at the BRB has been reported [13,14,15,16,17], and is suggested to be promising for the establishment of the safe and efficient delivery of cationic drugs to the retina where neurological dysfunctions are caused by severe retinal diseases such as macular degeneration and diabetic retinopathy [3,12]. In addition, lysosomal trapping has been implied to exert the undesirable influence on the blood-to-retina transport of cationic neuroprotectants across the BRB since its unignorable influence had been suggested in the accumulating reports [28,29,30]. The study using a representative lysosomotropic agent, LTR, suggested that lysosomal trapping only partially influenced transport of cationic amphiphilic drugs in the BRB [26], and this was also supported by the study using EFV [27]. In the present study, lysosome-enriched fraction was used to investigate the influence of lysosomal trapping on quinacrine and EFV uptake in the inner and outer BRB in detail. 

The study of quinacrine uptake by TR-iBRB2 cells showed an initial uptake rate of 445 µL/(min·mg protein), and suggested that quinacrine uptake was concentrated in the retinal capillary endothelial cells. This was because the quinacrine uptake at 30 min (1640 µL/[min·mg protein]) indicated that the cellular concentration of quinacrine was approximately 273 µM, which was clearly higher than that of the uptake buffer (5 µM) (Figure 1A). The uptake study at 4 °C showed that the quinacrine uptake was significantly reduced by 45% (Figure 1A), and this result implied the unignorable contribution of lysosomal trapping to quinacrine uptake in the inner BRB because the 45% reduction in cellular uptake of quinacrine seems to be insufficient to support the unilateral contribution of carrier-mediated transport. In addition, the experiment in TR-iBRB2 cells suggested that quinacrine uptake in the inner BRB occurs in an Na^+^- and membrane potential-insensitive manner (Figure 1B), and showed a sensitivity to extracellular pH. In addition, there was a 1.31-fold higher quinacrine uptake at pH 8.4 than at pH 7.4, whereas no significant change was observed in the uptake at pH 6.4 (Figure 1B). Based on the Henderson–Hasselbalch equation, the uptake of nonionized quinacrine at pH 8.4 was estimated to be 9.84-fold higher than that at pH 7.4, indicating that quinacrine uptake by TR-iBRB2 cells could not be explained by the pH-partition hypothesis. Furthermore, this observation suggests that passive diffusion in accordance with the physicochemical characteristics is not enough to explain the uptake of quinacrine in the inner BRB. This idea is closely supported by the estimation of K_m_, V_max_, and K_d_ values 1.87 μM, 189 μmol/(min·mg protein), and 29.7 μL/(min·mg protein), respectively, revealing the involvement of saturable and non-saturable processes in quinacrine uptake in the inner BRB (Figure 1C). Similarly, the transport was also reported to include saturable and non-saturable processes in the acidic intracellular compartments or at the plasma membrane of the inner BRB for the uptake of LTR, a typical lysosomotropic agent [26]. The results obtained for quinacrine and LTR demonstrate the difficulty involved in the study of lysosomal trapping using cellular uptake analysis, suggesting the possible advantage of using lysosomal fractions.

Bafilomycin A1 is a known specific inhibitor of V-ATPase, which is expressed in acidic intracellular compartments, including the lysosome, and contributes to maintaining their acidic interior pH [24]. Pretreatment with bafilomycin A1 significantly reduced quinacrine uptake by TR-iBRB2 cells (Figure 1D), suggesting that quinacrine uptake is largely influenced by the interior acidic pH in the inner BRB. In addition, the acute treatment of cells with NH_4_Cl or FCCP is known to cancel the H^+^-gradient between the lysosomes and cytosol [23,24,25], and TR-iBRB2 cells showed a significant decrease in quinacrine uptake following acute treatment with NH_4_Cl or FCCP, whereas pretreatment with NH_4_Cl had no effect (Figure 2A), supporting that the acidic interior pH is important in quinacrine uptake in the inner BRB. In addition to the saturable and non-saturable transport processes, the present results suggest that the characteristics of quinacrine transport were similar to those of LTR reported previously [26], and this was also supported by the results of the confocal microscopy because the fluorescence signals of quinacrine and LTR were shown to merge, suggesting quinacrine was distributed to the lysosome (Figure 2B). Confocal microscopy with bafilomycin A1 or NH_4_Cl also supported the sequestration of quinacrine in acidic intracellular compartments at the inner BRB because the punctate quinacrine distribution was shown to be attenuated in TR-iBRB2 cells treated with these agents (Figure 2C).

The inhibition study suggested that quinacrine possibly interacted with cationic amphiphilic drugs, except for clonidine, in the retinal capillary endothelial cells because the uptake of quinacrine by TR-iBRB2 cells was significantly reduced by desipramine, imipramine, propranolol, verapamil pyrilamine, and nicotine (Table 1). The study using bafilomycin A1 suggested that the interaction predominantly takes place in acidic intracellular compartments, including the lysosomes, and not at the plasma membrane, since TR-iBRB2 cells treated with bafilomycin A1 showed no significant change in quinacrine uptake in the presence of cationic drugs (Figure 2D). This observation clearly suggests the importance of studies with lysosomes in understanding the influence of lysosomal trapping on the transport of cationic drug at the BRB.

The uptake of quinacrine in the lysosome-enriched fractions of TR-iBRB2 cells was significantly inhibited by treatment with bafilomycin A1 (Figure 3A), and this was consistent with the results obtained in the cellular uptake with TR-iBRB2 cells (Figure 1D), supporting the distribution of quinacrine to lysosomes. The results of the inhibition study using several cationic drugs suggested that quinacrine interacted with desipramine, imipramine, propranolol and verapamil at the lysosome of the inner BRB, because quinacrine uptake in the lysosome-enriched fraction of TR-iBRB2 cells was significantly inhibited by these cationic drugs (Table 1). In addition, pyrilamine, clonidine, and nicotine had no effect on the uptake of quinacrine in the fractions of TR-iBRB2 cells (Table 1), and these results suggests that the interaction of these drugs in the lysosome is minor. These results also provide evidence that clearly supports previous reports of the carrier-mediated blood-to-retina transports of these agents across the inner BRB [13,14,15]. Similar results were also obtained with the lysosome-enriched fractions of RPE-J cells, used as an in vitro model cell line of the outer BRB (Table 1), and these results suggest the interaction of quinacrine with desipramine, imipramine, verapamil, and pyrilamine, and the blood-to-retina transport of clonidine and nicotine across the outer BRB.

Furthermore, the lysosome-enriched fraction of TR-iBRB2 cells was used to study the uptake of EFV, which was not changed by treatment with bafilomycin A1 (Figure 3B). This result was very similar to the outcome of our recent study of EFV uptake by TR-iBRB2 cells treated with bafilomycin A1 [27], and the similarity suggests that the interior acidic pH is not essential for EFV transport in the inner BRB (Figure 3B), showing that its transport characteristics differ from those of quinacrine. In the inhibition study, EFV uptake in TR-iBRB2 cell fractions was not altered in the presence of the cationic drugs (Table 2), suggesting a minor interaction between EFV and cationic drugs in lysosomes. The inhibition profile obtained was not consistent with that of quinacrine (Table 1 and Table 2), and these results suggests that the transport characteristics for EFV definitely differ from those of quinacrine in the inner BRB. These results also suggest the minor contribution of lysosomal trapping driven by acidic interior pH in EFV transport at the inner BRB, supporting the carrier-mediated blood-to-retina transport of EFV across the inner BRB [27]. In the lysosome-enriched fraction of RPE-J cells, EFV uptake was significantly inhibited by verapamil, whereas it was almost similar to those obtained in the fraction of TR-iBRB2 cells (Table 2 and Figure 3C). These results still suggest the clear difference between EFV transport characteristics and those of quinacrine in the outer BRB, supporting the fact that lysosomal trapping only partially influences EFV transport across the outer BRB as reported previously [27].

## 5. Conclusions

The cellular uptake study suggests that quinacrine was distributed to the acidic intracellular compartments of the inner BRB, and this was also supported by the confocal microscopy results. The inhibition of cellular uptake suggested the possible interaction of quinacrine with cationic drugs in lysosomes of the inner BRB, implying the importance of uptake studies in lysosomes. The adoption of the lysosome-enriched fraction in the uptake study suggested the lysosomal trapping of quinacrine in the BRB, and supported that lysosomal trapping exerts a minor influence on the transport of several cationic drugs at the BRB while its influence at varying degrees is also implied on the transport of other drugs. The study of EFV transport in lysosome-enriched fractions also suggested the minor influence of lysosomal trapping driven by interior acidic pH on the transport of EFV across the BRB. This observation also provides evidence supporting the blood-to-retina transport of verapamil across the BRB [15,17,27]. The present findings are expected to improve our understanding of the blood-to-retina transport of cationic amphiphilic drugs across the BRB, thereby contributing to the establishment of a new strategy for safe and efficient drug delivery to the retina.

## Figures and Tables

**Table 1:** Inhibitory Effect of Quinacrine Uptake.

Compounds		Relative Uptake (% of Control)										
		TR-iBRB2 Cells				Lysosome-Enriched Fraction of TR-iBRB2 Cells				Lysosome-Enriched Fraction of RPE-J Cells		
Control		100	±	3		100	±	3		100	±	11
Desipramine		47.9	±	2.2 **		47.2	±	1.9 **		64.4	±	0.9 **
Imipramine		55.2	±	2.7 **		49.6	±	5.0 **		61.8	±	2.9 **
Propranolol		55.4	±	2.5 **		65.8	±	1.8 **		76.8	±	3.8 *
Verapamil		64.7	±	1.2 **		42.4	±	1.3 **		57.3	±	2.1 **
Pyrilamine		72.6	±	2.3 **		87.1	±	5.0		66.0	±	2.4 **
Clonidine		90.3	±	6.6		106	±	11		89.4	±	6.8
Nicotine		79.5	±	6.0 **		106	±	2		97.1	±	7.1
Choline		98.7	±	3.3		N.D.				N.D.		
Cimetidine		88.3	±	1.9		N.D.				N.D.		
L-Carnitine		98.5	±	8.9		N.D.				N.D.		
PAH		97.6	±	8.2		N.D.				N.D.		

By using TR-iBRB2 cells and lysosome-enriched fractions, the uptake of quinacrine (300 nM) was examined in the presence of compounds (100 μM) for 30 min at 37 °C. Each value represents the means ± S.E.M. (*n* = 3–8). * *p* < 0.05, ** *p* < 0.01, significantly different from the control (absence of inhibitor). N.D., not determined. PAH, *p*-aminohippuric acid; N.D., not determined.

**Table 2:** Inhibitory effect of EFV uptake.

Compounds		Relative Uptake (% of Control)										
		TR-iBRB2 Cells [27]				Lysosome-Enriched Fraction of TR-iBRB2 Cells				Lysosome-Enriched Fraction of RPE-J Cells		
Control		100	±	3		100	±	3		100	±	7
Desipramine		47.9	±	2.2 **		61.8	±	1.9 *		65.5	±	5.8 *
Imipramine		55.2	±	2.7 **		68.5	±	2.8		82.5	±	11.0
Propranolol		55.4	±	2.5 **		108	±	13		114	±	7
Verapamil		64.7	±	1.2 **		71.5	±	3.0		51.0	±	3.8 **
Pyrilamine		72.6	±	2.3 **		143	±	1		122	±	12
Clonidine		90.3	±	6.6		132	±	7		98.6	±	4.1
Nicotine		79.5	±	6.0 **		147	±	23		110	±	10

By using TR-iBRB2 cells and lysosome-enriched fractions, the uptake of EFV (1 μM) by was examined in the presence of compounds (100 μM) at 37 °C for 3 min. Each value represents the means ± S.E.M. (*n* = 4). * *p* < 0.05, ** *p* < 0.01, significantly different from the control (absence of inhibitor). EFV, EverFluor FL Verapamil. Data for EFV uptake by TR-iBRB2 cells was presented by reference to previous report [27].

## Supplementary Materials

The Supplemental information are available online at https://www.mdpi.com/1999-4923/12/8/747/s1.
