# Supplementary material for: Uptake Study in Lysosome-Enriched Fraction: Critical Involvement of Lysosomal Trapping in Quinacrine Uptake but Not Fluorescence-Labeled Verapamil Transport at Blood-Retinal Barrier
Source: Pharmaceutics. 2020 Aug 8;12(8):747. doi: 10.3390/pharmaceutics12080747 (PMC7464812; doi:10.3390/pharmaceutics12080747)
Supplement: Supplementary file 1 [file pharmaceutics-12-00747-s001.pdf]

pharmaceutics

Supplementary Materials: Uptake Study in Lysosome-Enriched Fraction: Critical Involvement of Lysosomal Trapping in Quinacrine Uptake but Not Fluorescence-Labeled Verapamil Transport at Blood-Retinal Barrier

Supplemental information

Buffer contents

Phosphate-buffered saline (PBS)

137 mM NaCl, 2.7 mM KCl, 8.1 mM $Na_2HPO_4 \cdot 12H_2O$, 1.5 mM KH_2PO_4, pH 7.4

Extracellular fluid (ECF)-buffer

122 mM NaCl, 25 mM $NaHCO_3$, 3 mM KCl, 1.4 mM $CaCl_2$, 1.2 mM $MgSO_4$, 0.4 mM K_2HPO_4, 10 mM D-glucose and 10 mM HEPES, pH 7.4

Na^+-free ECF-buffer

122 mM LiCl, 25 mM $KHCO_3$, 3 mM KCl, 1.4 mM $CaCl_2$, 1.2 mM $MgSO_4$, 0.4 mM K_2HPO_4, 10 mM D-glucose and 10 mM HEPES, pH 7.4

ECF-buffer (pH6.4)

122 mM NaCl, 25 mM $NaHCO_3$, 3 mM KCl, 1.4 mM $CaCl_2$, 1.2 mM $MgSO_4$, 0.4 mM K_2HPO_4, 10 mM D-glucose and 10 mM MES, pH 6.4

ECF-buffer (pH8.4)

122 mM NaCl, 25 mM $NaHCO_3$, 3 mM KCl, 1.4 mM $CaCl_2$, 1.2 mM $MgSO_4$, 0.4 mM K_2HPO_4, 10 mM D-glucose and 10 mM Tris, pH 8.4

Cell Culture

TR-iBRB2 cells (passage number 23-35), a conditionally immortalized rat retinal capillary endothelial cell line, were cultured in Dulbecco's modified Eagle's medium (Nissui Pharmaceuticals, Tokyo, Japan) with 10% fetal bovine serum, 20 mM $NaHCO_3$, and antibiotics at 33°C under under an atmosphere of 5% CO_2 in air. The optimal growth temperature for TR-iBRB2 cells was set at 33°C because of the expression of temperature-sensitive large T-antigen in TR-iBRB2 cells.

The RPE-J cells (American Type Culture Collection, Manassas, VA, USA) were maintained in Dulbecco's modified Eagle's medium (Nissui Pharmaceuticals, Tokyo, Japan) with 4% fetal bovine serum, 20 mM $NaHCO_3$, 25 mM D-glucose, 0.1 mM nonessential amino acids, and antibiotics at 33°C under an atmosphere of 5% CO_2 in air. The RPE-J cells were cultured on Not TC–treated culture dishes (Corning, Corning, NY, USA).

Chemical Structure

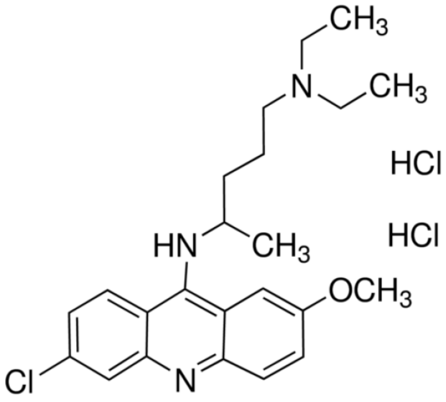

Quinacrine.

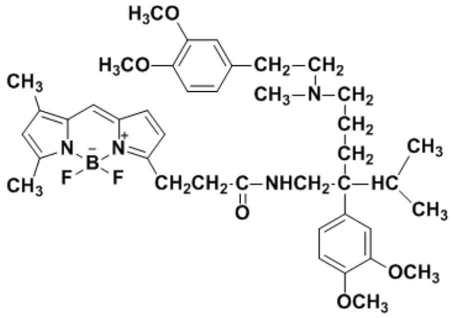

EverFluor FL Verapamil, (Known as BODIPY® FL Verapamil)

© 2020 by the authors. Licensee MDPI, Basel, Switzerland. This article is an open access article distributed under the terms and conditions of the Creative Commons Attribution (CC BY) license (http://creativecommons.org/licenses/by/4.0/).
